# A supervised machine learning statistical design of experiment approach to modeling the barriers to effective snakebite treatment in Ghana

**DOI:** 10.1371/journal.pntd.0012736

**Published:** 2024-12-13

**Authors:** Eric Nyarko, Edmund Fosu Agyemang, Ebenezer Kwesi Ameho, Louis Agyekum, José María Gutiérrez, Eduardo Alberto Fernandez

**Affiliations:** 1 Department of Statistics and Actuarial Science, College of Basic and Applied Sciences, University of Ghana, Legon, Accra, Ghana; 2 School of Mathematical and Statistical Science, College of Sciences, University of Texas Rio Grande Valley, Edinburg, Texas, United States of America; 3 Instituto Clodomiro Picado, Facultad de Microbiología, Universidad de Costa Rica, San José, Costa Rica; 4 Department of Health Sciences, Brock University, St Catharines, Ontario, Canada; Fundação de Medicina Tropical Doutor Heitor Vieira Dourado: Fundacao de Medicina Tropical Doutor Heitor Vieira Dourado, BRAZIL

## Abstract

**Background:**

Snakebite envenoming is a serious condition that affects 2.5 million people and causes 81,000–138,000 deaths every year, particularly in tropical and subtropical regions. The World Health Organization has set a goal to halve the deaths and disabilities related to snakebite envenoming by 2030. However, significant challenges in achieving this goal include a lack of robust research evidence related to snakebite incidence and treatment, particularly in sub-Saharan Africa. This study aimed to combine established methodologies with the latest tools in Artificial Intelligence to assess the barriers to effective snakebite treatment in Ghana.

**Method:**

We used a MaxDiff statistical experiment design to collect data, and six supervised machine learning models were applied to predict responses whose performance showed an advantage over the other through 6921 data points partitioned using the hold-back validation method, with 70% training and 30% validation. The results were compared using key metrics: Akaike Information Criterion corrected, Bayesian Information Criterion, Root Average Squared Error, and Fit Time in milliseconds.

**Results:**

Considering all the responses, none of the six machine learning algorithms proved superior, but the Generalized Regression Model (Ridge) performed consistently better among the candidate models. The model consistently predicted several key significant barriers to effective snakebite treatment, such as the high cost of antivenoms, increased use of unorthodox, harmful practices, lack of access to effective antivenoms in remote areas when needed, and resorting to unorthodox and harmful practices in addition to hospital treatment.

**Conclusion:**

The combination of a MaxDiff statistical experiment design to collect data and six machine learning models allowed the identification of barriers to accessing effective therapies for snakebite envenoming in Ghana. Addressing these barriers through targeted policy interventions, including intensified advocacy, continuous education, community engagement, healthcare worker training, and strategic investments, can enhance the effectiveness of snakebite treatment, ultimately benefiting snakebite victims and reducing the burden of snakebite envenoming. There is a need for robust regulatory frameworks and increased antivenom production to address these barriers.

## Author summary

Snakebite envenoming, a severe condition, affects 2.5 million people and causes 81,000–138,000 deaths annually, particularly in tropical and subtropical regions of Africa, Asia, and Latin America. The World Health Organization aims to reduce this burden by 50% by 2030. However, significant barriers exist to achieving these targets, especially in Sub-Saharan Africa. These barriers include limited rigorous research evidence and the lack of investment in effective antivenoms for local snake species. Meeting this goal will require innovative research to gather better data on snakebite incidence and treatment. Artificial intelligence is one promising field that can contribute to this effort. For the first time, we have demonstrated how MaxDiff statistical experiment designs and machine learning algorithms can be explored to predict the barriers to effective snakebite treatment, such as the high cost of antivenoms, increased use of harmful practices, lack of access to effective antivenoms in remote areas, and resorting to unorthodox and harmful practices in addition to hospital treatment. Addressing these barriers through targeted policy interventions, including intensified advocacy, continuous education, community engagement, healthcare worker training, and strategic investments, can enhance the effectiveness of snakebite treatment. Robust regulatory frameworks and increased local antivenom production are also needed to address these barriers.

## Introduction

Snakebite envenoming (SBE) is a Neglected Tropical Disease (NTD) and a severe condition that affects 2.5 million people [1], killing 81,000–138,000 individuals annually, including rural villagers, agricultural workers, pregnant women, young males, and working children, every year. It also disables another 400,000, mainly in tropical and subtropical Africa, Asia, and Latin America [1–5]. The World Health Organization (WHO) has launched a global strategy to reduce this burden of SBE by 50%, and to deliver three million effective treatments annually by 2030 [6]. However, significant barriers to achieving these targets in sub-Saharan Africa (SSA) include limited rigorous research evidence, misalignment of local priority needs, and the lack of investment towards the development and deployment of effective antivenoms targeting local snake species [2, 4, 6].

SBE represents a serious public health problem in Ghana, where a high incidence has been reported [7, 8]. In addition to the acute effects of envenoming, affected people are often left with permanent disabilities [9]. Therefore, improving information on SBE in Ghana through rigorous research evidence is crucial to improve prevention and treatment. This will create a stronger foundation for developing strategies that meet local needs, aligning with the WHO’s goal of reducing the burden of SBE. Achieving this will involve innovative research by expanding the traditional methods used in health research, modifying existing methodologies, and integrating new tools from various fields to create new solutions [10]. Artificial Intelligence (AI), including Machine Learning (ML) and deep learning, is one promising field that can contribute to this effort by simulating human analytical skills for problem-solving.

Based on recent technological advancements, ML algorithms are now being used in various contexts on a larger scale than ever before. As a result, AI and ML are seen as innovations that will significantly impact our work methods and organization in the near future [11]. ML algorithms are typically used in situations where there is abundant data available, and by analyzing such large datasets, the algorithms can identify the underlying patterns or trends that describe the studied phenomenon, thus establishing connections between the relevant variables [12]. This process is called the algorithm’s ‘learning’ or ‘training’ phase. It is important to note that this approach is mainly empirical, and the results’ robustness and validity directly depend on the exhaustiveness of the dataset selected for training the algorithms.

ML tools are valuable in clinical decision-making, offering real-time insights and recommendations based on extensive data. These tools can help clinicians choose the most effective treatments, dosages, and care plans tailored to individual patient needs. For instance, ML-driven decision support systems can assist emergency departments in triaging patients more efficiently, thereby reducing waiting times and improving the allocation of medical resources [13]. The available research in SBE that utilizes AI, including ML and deep learning, primarily focuses on snake identification [14–18]. However, in line with WHO’s global strategy to halve the burden of SBE by 2030, it is essential to apply innovative research methods to better understand the underlying phenomena associated with this disease. This means expanding the classic health research toolkit by modifying existing methodologies and integrating new tools to gather better snakebite information. Doing so can create a stronger foundation for developing strategies to combat this NTD.

In recent years, combining data collected through statistical experiment design strategies with ML models has found application in diverse fields such as manufacturing, chemical, quality improvement, and biomedical industries [12,19,20]. ML algorithms are used because of their innovativeness and potentially high predictive power when compared with the most common classic models employed to model relationships of data generated from statistical experiment designs [12]. Although based on correlations, the relationships revealed by the models can offer valuable insights into causation through the combined use of statistical experiment design, indicating that the models can achieve prediction and inference. This study aims to explore the combined use of MaxDiff statistical experiment designs and different ML algorithms in the setting of SBE. Six supervised ML models—Support Vector Regression (SVR), Generalized Regression Model with Lasso, Pruned Forward Selection, Forward Selection, Elastic Net, and Ridge Regularization—are considered, and the resultant predictive performance to the barriers to effective snakebite treatment are compared. We show for the first time that ML algorithms and MaxDiff statistical experiment designs can be explored in SBE to accurately predict the barriers to effective snakebite treatment. This research is essential as it addresses a significant public health issue in Ghana, where SBE causes considerable morbidity and mortality, especially in rural areas with limited healthcare resources. The findings of this study offer evidence-based insights to help healthcare professionals and researchers understand how well ML algorithms can predict barriers to effective snakebite treatment. This information can shape healthcare policies, improve the distribution and usage of antivenoms, and ultimately enhance the effectiveness of snakebite treatment and improve the quality of patient care in Ghana and other sub-Saharan African regions. This, in turn, can help reduce the burden of SBE.

## Methods

### Ethics statement

The study received ethical approval from the Ethics Committee of the College of Basic and Applied Sciences, University of Ghana (Reference No: ECBAS 065/22–23) and adhered to all ethical guidelines and regulations. After explaining the purpose of the study to all participants, written informed consent was obtained. Participants were informed that their participation was voluntary and that they had the option to choose whether to participate in the study or not.

### MaxDiff design

**Fig 1:**
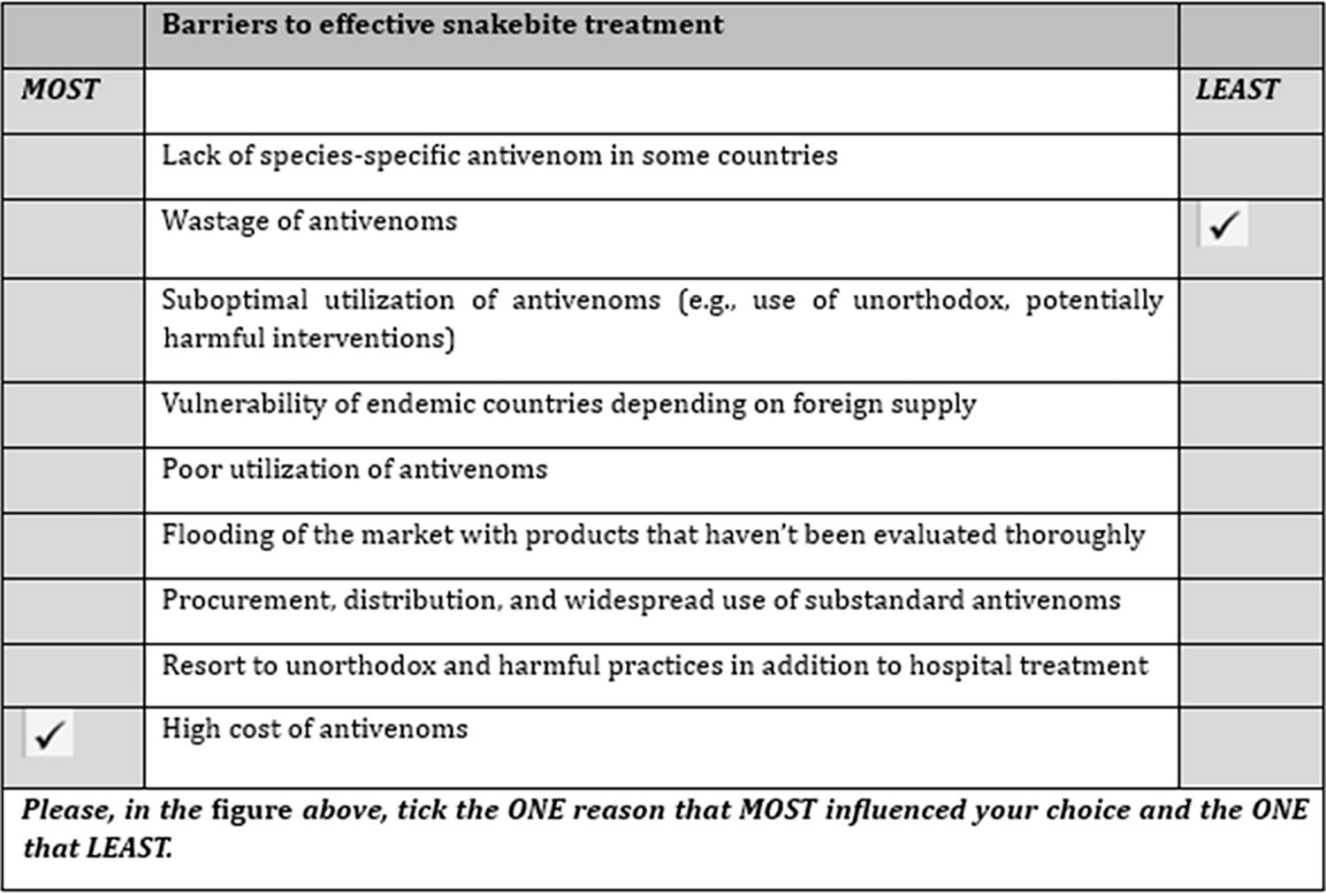
A sample completed scenario presented to respondents. The set of scenarios generated for this study contain nine barriers to effective snakebite treatment each, and the sample completed scenario represents the third best-worst scaling scenario labelled as A9, A19, A16, A18, A11, A2, A12, A14 and A3.

We utilize an available MaxDiff design data set previously obtained from a cross-sectional study conducted in August 2023 among a simple random sample of 203 healthcare workers, including community health nurses, physician assistants/medical doctors, and certificate/enrolled/general nurses in Kwahu Afram Plains North and South Districts of the Eastern Region, Ghana [21]. The healthcare workers were employed in health facilities such as community-based health planning and services compounds, health centers, clinics, and hospitals. The design considered 19 attributes related to the barriers to effective snakebite treatment based on focus group discussions with healthcare workers and a literature search [2,21,22]. A statistical experiment block design [23] was used to generate 19 choice scenarios, each with nine attributes for the survey, as shown in Fig 1. The attributes used in the study are as follows: (A1) Flooding of market with substandard antivenoms, (A2) Flooding of the market with products that haven’t been evaluated thoroughly, (A3) High cost of antivenoms, (A4) Inadequate doses to address need, (A5) Inadequate number of manufacturers, (A6) Increased use of unorthodox, harmful practices, (A7) Lack of access to effective antivenoms in remote areas when needed, (A8) Lack of effective antivenoms to address envenoming from local species in some instances, (A9) Lack of species-specific antivenom in some countries, (A10) Poor outcomes from use of substandard antivenoms, (A11) Poor utilization of antivenoms, (A12) Procurement, distribution, and widespread use of substandard antivenoms, (A13) Resort to substandard, cheap, and harmful antivenoms, (A14) Resort to unorthodox and harmful practices in addition to hospital treatment, (A15) Stock outs, (A16) Suboptimal utilization of antivenoms (e.g., use of unorthodox, potentially harmful interventions), (A17) Use of ineffective, substandard antivenoms, (A18) Vulnerability of endemic countries depending on foreign supply, and (A19) Wastage of antivenoms.

Similar to this study, in MaxDiff design, respondents are asked to choose the best (or most desired) and worst (or least desired) items from choice sets of size three or more [21,24]. MaxDiff design was selected in the previous study as the appropriate methodology for data collection for the following reasons: it offers a key advantage in gauging the relative importance of all attributes on a standardized scale. Unlike traditional rating scale surveys, this method demands a higher level of participant engagement and cognitive effort, potentially aiding in maintaining focus during the decision-making process [25]. Moreover, its appeal over discrete choice experiments has been highlighted in the literature [26,27].

The inclusion criteria for this study were community healthcare workers who had received training in snakebite management, had significant experience and had been involved in treating snakebite victims at their facilities. These workers should have administered antivenoms or encountered snakebite victims and provided their consent to participate in the study. Conversely, community healthcare workers who lacked experience, had never managed snakebite victims in their facilities, had not encountered snakebite victims, or did not provide consent were excluded from this study.

### Data preparation for model building

In this paper, in which the focus consists of the joint application of MaxDiff design and ML algorithms to predict the barriers to effective snakebite treatment, the first step in this process is data collection. After gathering the data, a model must be developed to identify relationships within the data and predict a specific response of interest. The dataset collected from the experiment revealed no issues related to outliers with extreme or impossible responses or problems with missing data. The study ensured that no data was missing because it used an interviewer-administered questionnaire. Enumerators were present to conduct initial checks for any missing values in the survey tools and encouraged respondents to complete any incomplete sections. As a result, all 203 distributed questionnaires were included in the final analysis.

The final MaxDiff design data set was partitioned using the hold-back validation method [28], with 70% training for model fitting and 30% validation for performance evaluation. One common way to prevent overfitting (where the model learns not only the intended patterns but also some of the noise in the training data, impacting its performance on new data) is to hold back part of the training data for validation [29]. It is important to note that the predictor variables, which represent the barriers to effective snakebite treatment, were considered generic attributes and the response variable was considered continuous. In this study, the MaxDiff design data set was analyzed using six supervised ML algorithms based on the barriers to effective snakebite treatment.

### Machine learning models

We utilized supervised ML algorithms with continuous response variables to predict the barriers to effective snakebite treatment based on the MaxDiff design data set. Six ML models are chosen as candidates: Support Vector Regression (SVR), Generalized Regression Model with least absolute shrinkage and selection operator (LASSO), Pruned Forward Selection, Forward Selection, Elastic Net, and Ridge regularization. These models were validated internally using the same data source on which they were developed.

### Generalized Regression (LASSO) model

Lasso is a regression analysis method that performs both variable selection and regularization [30]. The objective function is given by

$$\underset{\beta }{\min}\left\{\frac{1}{2n}\sum_{i=1}^{n}{\left(y_{i}-x_{i}^{T}\beta \right)}^{2}+\lambda |\beta {|}_{1}\right\},$$ (1)

where *n* is the total number of observations, *y*_*i*_ is the response variable, ***x***_*i*_ is the vector of predictor variables, ***β*** is the vector of coefficients, *λ* is regularization parameter, and |***β***|_1_ is the L1 norm of the coefficients, promoting sparsity in the model by preventing overfitting through shrinking the model coefficients toward zero and performing variable selection.

### Generalized Regression (Ridge) model

Moreover, the Ridge regression adds an L2 penalty to the least squares objective function [31]

$$\underset{\beta }{\min}\left\{\frac{1}{2n}\sum_{i=1}^{n}{\left(y_{i}-x_{i}^{T}\beta \right)}^{2}+\lambda |\beta {|}_{2}^{2}\right\},$$ (2)

where $|\beta {|}_{2}^{2}$ is the L2 norm of the coefficients. This also helps prevent overfitting by shrinking the coefficients toward zero but does not perform variable selection.

### Generalized Regression (Elastic Net) model

The Elastic Net is a regularization technique that combines the Lasso and Ridge regression properties [32]. Its objective function is given by

$$\underset{\beta }{\min}\{\frac{1}{2n}\sum_{i=1}^{n}{\left(y_{i}-x_{i}^{T}\beta \right)}^{2}+\lambda_{1}|\beta {|}_{1}+\lambda_{2}|\beta {|}_{2}^{2}\},$$ (3)

where |***β***|_1_ is the L1 norm and $|\beta {|}_{2}^{2}$ is the L2 norm of the model coefficients. This method retains the benefits of both L1 and L2 regularization, ensuring sparsity and grouping of correlated variables. Pruned Forward Selection, which is a stepwise regression method, sequentially adds predictor variables to the model. The variable that provides the most significant improvement in the model fit is added at each step. Here, the process continues until no further improvement is observed [33].

### Support Vector Regression (SVR)

SVR was employed in this study to find a function that approximates the mapping from input features to continuous outputs while minimizing the prediction error [34]. Its objective function is given by

$$\underset{w,b,\xi ,\xi^{*}}{\min}\left\{\frac{1}{2}|w{|}^{2}+C\sum_{i=1}^{n}\left(\xi_{i}+\xi_{i}^{*}\right)\right\}$$ (4)

subject to the constraints:

$$y_{i}-(w^{T}\phi (x_{i})+b)\leq \epsilon +\xi_{i},$$

$$(w^{T}\phi (x_{i})+b)-y_{i}\leq \epsilon +\xi_{i}^{*},$$ (5)

$$\xi_{i},\xi_{i}^{*}\geq 0,\;\forall i=1,\ldots ,n,$$

where ***w*** is the weight vector, *b* is the bias term, *ξ*_*i*_ and $\xi_{i}^{*}$ are slack variables, *C* is the regularization parameter, *ξ* is the margin of tolerance, and *ϕ* represents the kernel function. In this study, we used the radial basis function (RBF) kernel:

$$\phi (x_{i},x_{j})=\exp\left(-\gamma |x_{i}-x_{j}{|}^{2}\right),$$ (6)

where *γ* is a parameter that determines the width of the RBF kernel. The parameters *C* and *γ* were tuned using grid search to optimize the performance of the SVR model. For the grid search, we set up the parameters and the distributions to sample and fine-tune the hyperparameters by setting the cost or regularization parameter at 0.1, 1, 10, 100, and the kernel coefficients at 1, 0.1, 0.01, and 0.001. After the search, the best kernel coefficient, *γ*, resulted in 1 with a regularization parameter of 1. These were used in the RBF kernel for the model.

### Generalized Regression (Forward Selection) model

In the pursuit of enhancing predictive accuracy and model interpretability in ML applications, forward selection has emerged as a pivotal technique [35]. This method is particularly advantageous in the context of our research on barriers to effective snakebite treatment, where the complexity and multicollinearity of variables may necessitate robust variable selection mechanisms. Pruned selection is a process used in ML to improve model performance and reduce overfitting by removing nonsignificant variables (or attributes). This method systematically evaluates each predictor variable and removes those that do not contribute significantly to the model’s predictive power. Specifically, for the forward selection, variables are added to the model one at a time based on their statistical significance. The process begins with no variables in the model, and at each step, the variable that provides the most significant improvement to the model’s fit is added. The goal is to simplify the model while maintaining or improving its accuracy.

### Generalized Regression (Pruned Forward Selection) model

Pruned forward selection [35] is a specific type of stepwise regression that combines the principles of forward selection with pruning. This method goes a step further by including a pruning mechanism. After adding a new variable, the method also considers removing variables that have become non-significant due to the inclusion of the new variable. This iterative process of adding and removing variables continues until no significant improvement can be achieved.

### Statistical analysis of data

All statistical analyses involving the six ML algorithms were performed using JMP Pro (Version 17.0). The final data set, including 6921 data points, was partitioned using the hold-back validation method, with 70% training and 30% validation. The results are compared using several key metrics: Akaike Information Criterion corrected (AICc), Bayesian Information Criterion (BIC), Root Average Squared Error (RASE), and Fit Time in milliseconds. These metrics help determine the best-performing model by assessing the trade-offs between goodness of fit, model complexity, and computational efficiency. Lower AICc and BIC values indicate a better fit relative to the number of parameters used. Similarly, a lower RASE value signifies the best-performing model. We evaluated the significance of the barriers to effective snakebite treatment at the 95% confidence intervals (CIs). A parameter estimate (***β***) was also deemed statistically significant if the significant probability (*p*-value) was either less than or equal to 0.001, 0.01, and 0.05. The parameter estimate’s sign indicates whether the attribute has a positive or negative utility effect.

## Results

### Model fit and overall performance

Among the models evaluated, the Generalized Regression (Ridge) model stands out with intermediate AICc and BIC values of 6112.384 and 6241.919, respectively. Lower values of AICc and BIC indicate a better fit relative to the number of parameters used, suggesting that the Ridge model effectively balances model complexity and goodness of fit. Additionally, the Ridge model exhibits an intermediate RASE value of 0.452, implying higher prediction accuracy than other candidate models. Relatively, the Generalized Regression (Forward Selection) and (Elastic net) models exhibit higher performance with AICc values of 6090.869 and 6040.900, BIC values of 6220.405 and 6170.436, and slightly higher RASE values of 0.454 and 0.459, respectively. Regarding fit time, the Generalized Regression (Forward Selection) model is the most efficient, with a fit time of just 124 milliseconds. However, it is important to note that this model has a slightly higher RASE value, indicating a trade-off between computational efficiency and predictive performance. The Generalized Regression (Forward Prune Selection) and (Lasso) models exhibit lower goodness of fit with AICc values of 6158.199 and 6141.509, BIC values of 6287.734 and 6264.575, but higher prediction accuracy of RASE values of 0.446 and 0.448, with fit times of 147 and 2313 milliseconds, respectively. The Support Vector Regression (RBF kernel) model, while offering the lowest predictive performance with a RASE of 0.461, also has the longest fit time at 9374 milliseconds, making it less desirable for applications where computational efficiency and predictive performance are critical. Overall, the Generalized Regression (Ridge) model is the best candidate model, achieving general intermediate AICc, BIC, and RASE values, indicating superior model fit and predictive accuracy despite having a higher fit time of 2756 milliseconds compared to the other candidate models except for the SVM. Its overall performance justifies the computational cost, making it the preferred choice for applications prioritizing accuracy and model reliability.

### Prediction profilers for each type of ML model

**Fig 2:**
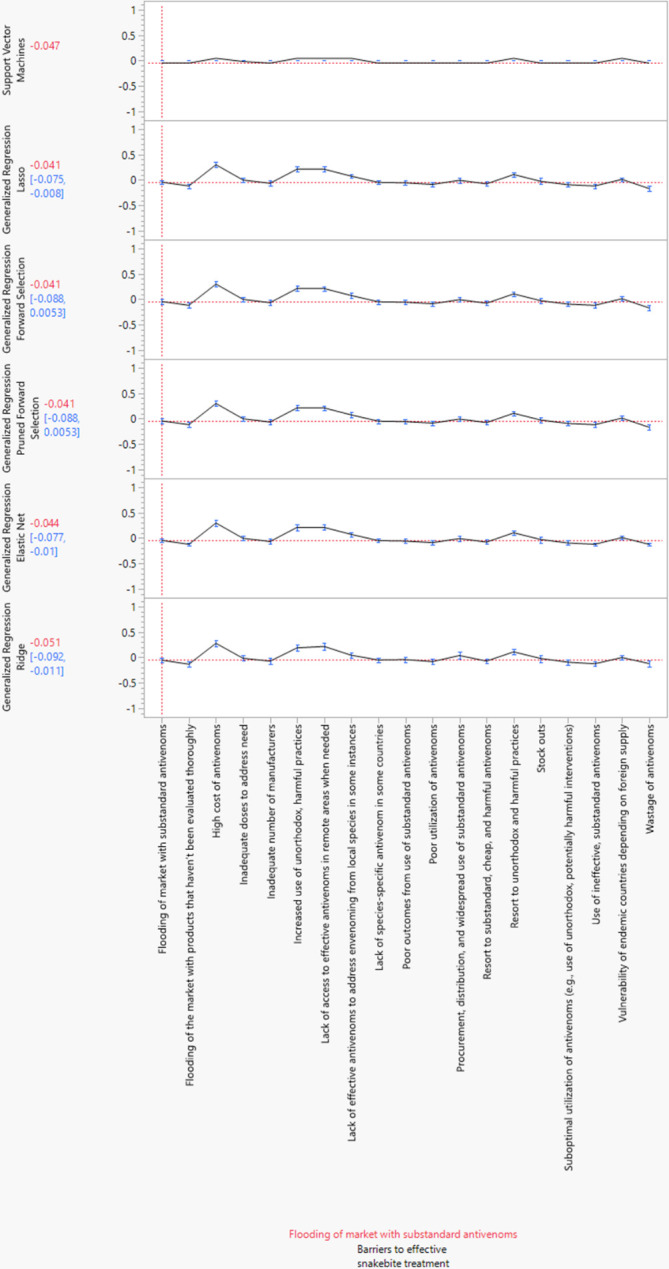
Prediction profiler for barriers to effective snakebite treatment.

The utility profilers for the six ML algorithms are exhibited in Fig 2. The utility profiler is used to visualize the importance of the barriers to effective snakebite treatment. The vertical red line of the utility profiler corresponds to the baseline/reference barrier. Only the individual barriers to effective snakebite treatment with CIs above the horizontal red lines of the profilers are significantly different at 95%CIs. Relative to the flooding of the market with substandard antivenoms, the ML algorithms generally predict (A3) High cost of antivenoms, (A4) Inadequate doses to address need, (A6) Increased use of unorthodox, harmful practices, (A7) Lack of access to effective antivenoms in remote areas when needed, (A8) Lack of effective antivenoms to address envenoming from local species in some instances, (A12) Procurement, distribution, and widespread use of substandard antivenoms, (A14) Resort to unorthodox and harmful practices in addition to hospital treatment, (A15) Stock outs and (A18) the Vulnerability of endemic countries depending on foreign supply as significant barriers to effective snakebite treatment.

### Generalized Regression (Ridge) model

Table 1. presents the best-performing candidate model results. Relative to the baseline attribute, the model predicts several key significant barriers to effective snakebite treatment, such as (A3) High cost of antivenoms (***β*** = 0.394, 95%CI: 0.303, 0.486), (A4) Inadequate doses to address need (***β*** = 0.101, 95%CI: 0.019, 0.182), (A6) Increased use of unorthodox, harmful practices (***β*** = 0.306, 95%CI: 0.214, 0.398), (A7) Lack of access to effective antivenoms in remote areas when needed (***β*** = 0.333, 95%CI: 0.244, 0.422), (A8) Lack of effective antivenoms to address envenoming from local species in some instances (***β*** = 0.161, 95%CI: 0.082, 0.239), (A12) Procurement, distribution, and widespread use of substandard antivenoms (***β*** = 0.154, 95%CI: 0.059, 0.249), (A14) Resort to unorthodox and harmful practices in addition to hospital treatment (***β*** = 0.227, 95%CI: 0.146, 0.308), (A15) Stock outs (***β*** = 0.096, 95%CI: 0.004, 0.189), and (A18) Vulnerability of endemic countries depending on foreign supply (***β*** = 0.116, 95%CI: 0.042, 0.189). It can be inferred that the high cost of antivenoms is the highest barrier to effective snakebite treatment, followed by lack of access to effective antivenoms in remote areas when needed, increased use of unorthodox, harmful practices, resort to unorthodox and harmful practices in addition to hospital treatment, lack of effective antivenoms to address envenoming from local species in some instances, procurement, distribution, and widespread use of substandard antivenoms, vulnerability of endemic countries depending on foreign supply, inadequate doses to address need, as well as stock outs. Addressing these key barriers to effective snakebite treatment is essential for developing snakebite management, antivenom regulation, procurement and supply strategies that meet local needs and help achieve the WHO’s goal of halving the global burden of snakebite envenoming by 2030.

**Table 1:** Generalized Regression model with Ridge results of the barriers to effective snakebite treatment.

Attribute	Estimate	Std Error	Wald ChiSquare	Prob*>*ChiSquare	Lower 95%	Upper 95%
A1	0.064	0.037	2.960	0.085	-0.009	0.138
A2	-0.014	0.040	0.130	0.717	-0.093	0.064
A3	0.394	0.046	71.550	0.000*	0.303	0.486
A4	0.101	0.041	5.911	0.015*	0.019	0.182
A5	0.045	0.043	1.094	0.295	-0.040	0.131
A6	0.306	0.046	42.649	0.000*	0.214	0.398
A7	0.333	0.045	54.014	0.000*	0.244	0.422
A8	0.161	0.040	16.153	0.000*	0.082	0.239
A9	0.067	0.038	3.065	0.080	-0.008	0.143
A10	0.075	0.039	3.682	0.055	-0.001	0.153
A11	0.036	0.042	0.736	0.390	-0.046	0.119
A12	0.154	0.048	10.064	0.001*	0.059	0.249
A13	0.046	0.040	1.362	0.243	-0.031	0.125
A14	0.227	0.041	30.313	0.000*	0.146	0.308
A15	0.096	0.047	4.195	0.040*	0.004	0.189
A16	0.025	0.041	0.378	0.538	-0.055	0.107
A17	-0.006	0.040	0.021	0.882	-0.085	0.073
A18	0.116	0.037	9.576	0.002*	0.042	0.189
**Model Fit Statistics**						
AICc	6112.384					
BIC	6241.919					
RASE	0.452					
p-value	0.000*					

## Discussion

In this paper, we unveiled a novel application of ML algorithms and MaxDiff statistical experiment design in the context of SBE. For the first time, we demonstrated how these tools can be used to predict the barriers to effective snakebite treatment. The ML model results should be highly relevant to clinicians, researchers, antivenom producers, policymakers, and everyone involved in antivenom management, procurement, supply, and supporting systems to improve outcomes for snakebite victims. Identifying the critical barriers to effective snakebite treatment is crucial for developing strategies that address local needs and contribute to the WHO’s goal of reducing the global burden of SBE by 50% by 2030 [6]. Our exploration involved six supervised ML algorithms—Support Vector Regression (SVR), Generalized Regression Model with LASSO, Pruned Forward Selection, Forward Selection, Elastic Net, and Ridge Regularization—to compare their goodness of fit and predictive performance on the barriers to effective snakebite treatment.

The MaxDiff statistical experiment design and data analysis using ML techniques represent an innovative and advanced research approach. This method is particularly advantageous because it can leverage high-quality data from carefully constructed experiment setups, resulting in robust foundational inferences in practice.

With data collected in accordance with the guidelines of the MaxDiff statistical experiment technique, the suggested framework instills a sense of enhanced confidence in the caliber of the dataset used to train the ML models, providing a strong foundation for model development. The ML algorithms scrutinized the dataset obtained from the MaxDiff statistical experiment design. This rigorous process aimed to unveil and understand the intricate relationships within the data, enabling the prediction of barriers to effective snakebite treatment. Additionally, ML offers the capability to model complex relationships with minimal assumptions regarding the MaxDiff statistical experiment design data.

Furthermore, the judicious selection of ML algorithms can be applied to MaxDiff statistical experiment design data that allows for improved predictive performance without significant loss in terms of interpretability with respect to the classical candidate model. Accordingly, in the six ML models developed for predicting the barriers to effective snakebite treatment, the results were generally satisfactory regarding accuracy, reliability, and interpretability. Considering our results, none of the six ML algorithms proved superior, but they all performed consistently better than the classical maximum difference model [21].

The Generalized Regression (Ridge) model performed consistently better among the ML candidate models. This model consistently predicted several significant key barriers to effective snakebite treatment, such as the high cost of antivenoms, increased use of unorthodox, harmful practices, lack of access to effective antivenoms in remote areas when needed, and resorting to unorthodox and harmful practices in addition to hospital treatment. These findings are consistent with previous studies that explored the existing limitations for effective treatment of snakebite envenoming [21, 36–39]. Addressing these key barriers to effective snakebite treatment is essential for developing snakebite management, antivenom regulation, procurement and supply strategies that meet local needs and help achieve the WHO’s goal of halving the global burden of snakebite envenoming by 2030 [6]. There is a need for robust regulatory frameworks and increased antivenom production to address these barriers [3, 22]. Further studies should explore these limitations in specific contexts in Ghana and elsewhere in sub-Saharan Africa.

This research shows for the first time that ML algorithms and MaxDiff statistical experiment design have been used in SBE to predict the barriers to effective snakebite treatment. Nevertheless, it is important to acknowledge the limitations of this research. The results obtained from the study may have broader implications for other regions, as the probability sampling technique allows for generalizability. However, it is essential to note that the study covered a limited geographical area. Further work is needed to determine whether there are significant regional differences in Ghana between regions in terms of cultural and public health issues. Furthermore, this study was based on the views of healthcare workers. Future advanced studies should consider the perspectives of other stakeholders related to snakebite envenoming, such as community members and traditional healers [39, 40], to further expand the landscape of the analysis of this complex public health issue.

The approach taken in this study, while primarily based on ML models, cannot be categorized as solely empirical or based purely on black-box algorithms. Utilizing tools already present in the literature, the black box is illuminated, and information is provided regarding the significance of each barrier to effective snakebite treatment within the models. It is also essential to remember that the data used to develop the models is derived from statistical experiment design. Therefore, although based on correlations, the relationships the ML models reveal can offer valuable insights into causation, indicating that the models can achieve prediction and inference [12].

In conclusion, we have demonstrated that if the objective is to generate strong conclusions on crucial barriers to effective snakebite treatment, ML models should not only provide accurate predictions but should also offer insights into their interpretability to enhance understanding and create a solid foundation for policy discussions on SBE. At this point, the ML models learned, in particular the Generalized Regression (Ridge) model, can be used in practice to identify barriers to effective snakebite treatment across different areas of the sub-region. These models provide new insights, such as the most common barriers in specific regions, the factors contributing to these barriers, and potential solutions to overcome them. These insights have the potential to significantly influence healthcare policies, optimize the procurement, supply, and use of antivenoms, and ultimately improve both the effectiveness of snakebite treatment and the quality of patient care in Ghana and other sub-Saharan African regions.

## Supporting information

S1 Data Cs: consideration sets, ppt: facilities, x_1, x_12, x_13, x_14, x_15: generated design matrix. (XLSX)
